# Expression profiles of α-synuclein in cortical lesions of patients with FCD IIb and TSC, and FCD rats

**DOI:** 10.3389/fneur.2023.1255097

**Published:** 2023-11-07

**Authors:** Li Zhang, Jun Huang, Lu Dai, Gang Zhu, Xiao-Lin Yang, Zeng He, Yu-Hong Li, Hui Yang, Chun-Qing Zhang, Kai-Feng Shen, Ping Liang

**Affiliations:** ^1^Department of Neurosurgery, Children's Hospital of Chongqing Medical University, National Clinical Research Center for Child Health and Disorders, Ministry of Education Key Laboratory of Child Development and Disorders, Chongqing Key Laboratory of Pediatrics, Chongqing, China; ^2^Department of Neurosurgery, Epilepsy Research Center of PLA, Xinqiao Hospital, Army Medical University, Chongqing, China; ^3^Chongqing Institute for Brain and Intelligence, Guang Yang Bay Laboratory, Chongqing, China; ^4^Department of Cell Biology, Basic Medical College, Army Medical University, Chongqing, China

**Keywords:** FCD IIb, TSC, FCD rats, α-syn, NMDAR

## Abstract

**Background:**

Focal cortical dysplasia (FCD) IIb and tuberous sclerosis complex (TSC) are common causes of drug-resistant epilepsy in children. However, the etiologies related to the development of FCD IIb and TSC are not fully understood. α-synuclein (α-syn) is a member of synucleins family that plays crucial roles in modulating synaptic transmission in central nervous system. Here, we explored the expression profiles and potential pathogenic functions of α-syn in cortical lesions of epileptic patients with FCD IIb and TSC.

**Methods:**

Surgical specimens from epileptic patients with FCD IIb and TSC, as well as FCD rats generated by *in utero* X-ray-radiation were adopted in this study and studied with immunohistochemistry, immunofluorescence, western blotting, and co-immunoprecipitation etc. molecular biological techniques.

**Result:**

Our results showed that α-syn expression was reduced in FCD IIb and TSC lesions. Specifically, α-syn protein was intensely expressed in dysplastic neurons (DNs) and balloon cells (BCs) in FCD IIb lesions, whereas was barely detected in DNs and giant cells (GCs) of TSC lesions. Additionally, p-α-syn, the aggregated form of α-syn, was detected in DNs, BCs, GCs, and glia-like cells of FCD IIb and TSC lesions. We previous showed that the function of N-methyl-D-aspartate receptor (NMDAR) was enhanced in FCD rats generated by X-ray-radiation. Here, we found the interaction between α-syn and NMDAR subunits NMDAR2A, NMDAR2B were augmented in cortical lesions of FCD patients and FCD rats.

**Conclusion:**

These results suggested a potential role of α-syn in the pathogenesis of FCD IIb and TSC by interfering with NMDAR.

## 1. Introduction

Malformations of cortical development (MCDs), including focal cortical dysplasia (FCDs) and tuberous sclerosis complex (TSC), are the most common pathological causes of drug-resistant epilepsy in children (1). FCDs were classified into FCD type I, IIa, and IIb according to the current international consensus classification established by the International League against Epilepsy (ILAE). Type I FCD is characterized histologically with microcolumnar neurons, and type II is characterized by cortical dyslamination and dysplastic neurons (DNs) with (Type IIb) or without balloon cells (BCs) (Type IIa) (2). Similar to the pathological characteristics of FCD IIb, TSC lesions are characterized with DNs and giant cells (GC) under pathological examination (3). Plentiful investigations were conducted to reveal the etiology of FCD IIb and TSC; however, present studies are still insufficient to elucidate the pathological and epileptic mechanisms of FCD IIb and TSC.

α-synuclein (α-syn), a member of the synuclein family, plays various routes to cellular dysfunction by inducing synaptic dysfunction, mitochondrial impairment, defective endoplasmic reticulum (ER) function, autophagy–lysosomal pathway, and nuclear dysfunction (4). α-syn widely localizes in presynaptic terminals as a monomer and plays crucial roles in regulating neurotransmitter release, synaptic function, and plasticity in the central nervous system (5). Additionally, studies have shown that α-syn monomers tend to aggregate in structures of higher molecular weights leading to the formation of α-syn oligomers, protofibrils, and eventually fibrils, the main components of Lewy bodies in neurodegenerative diseases (6). Thus, the versatile roles of α-syn in mediating cellular function intrigue us on whether it plays important roles in the etiology of FCD IIb and TSC.

Due to ethical limitations, many investigations cannot be carried out in humans. To address the etiology and pathogenesis of FCD, many animal models of FCD have been established to investigate the mechanisms of epileptogenesis and novel therapeutics for epilepsy. Historically, chemical or physical induction during early life was adopted to create the FCD model in animals, including neonatal freeze lesions (7, 8) or *in utero* irradiation to generate microgyria and focal heterotopia (9–11), *in utero* exposure to methylazoxymethanol (MAM) acetate (12–17), carmustine 1–3-bis-chloroethyl-nitrosourea (BCNU) (18), and neonatal exposure ibotenic acid to generate focal cortical malformations (19). Transgenic and *in utero* electroporation-based animal models were used to target genes of the mTOR pathway and recapitulate, to various degrees, the cortical malformations and recurrent spontaneous seizures in recent years (20–22). However, there is no “perfect” animal model as no single model recapitulates all the human phenotypes associated with FCD. Rat models are more economic and easier to establish, e.g., compared with the ferret model (14–17). When compared with mice, rats are larger in size. More importantly, rats are closer to humans with less genomic differences and greater physiological similarities in consequence, making them the ideal and classical animal for constructing FCD models (23–26), whereas chemical or physical stimulation-induced mouse models of FCD have been rarely reported. In the study, we used *in utero* irradiation of pregnant rats to X-ray to generate FCD rats in consistent with the procedures used in our previous study (27). Striking the loss of normal six-layered cortex, varying degrees of cytomegalic or dysmorphic cells and spontaneous seizures were reported in these animals (9, 28, 29).

Thus, to explore the potential function of α-syn in the pathogenesis of FCD IIb and TSC, we conducted a study using surgical specimens from epileptic patients with FCD IIb and TSC, as well as FCD rats generated by *in utero* X-ray-radiation, to investigate the expression patterns of α-syn in cortical lesions and the interplay of α-syn and N-methyl-D-aspartate receptor (NMDAR), which is the primary receptor mediating excitatory synaptic transmission in central nervous system.

In the study, we found that α-syn was reduced, whereas the interaction between α-syn and NMDAR was strengthened in the cortical lesions of patients with FCD IIb and TSC, suggesting a complex function of α-syn in the pathogenesis of FCD. Cortical dyslamination and thinning were the most prominent histological abnormities in FCD rats during our observation from 7 to 28 days, in parallel with the alteration of α-syn and NMDAR in the cellular level, which highly simulated the pathological features of human FCD and suggested the disruption of brain function, including epileptogenesis in FCD rats. Immunotherapeutic approaches have been used to reduce α-syn pathology in rodent models with neurodegenerative diseases. However, many challenges existed before achieving an effective treatment strategy, including the overcoming of the blood–brain barrier and the complex heterogeneity of α-syn (30). Similar progresses have been achieved in unraveling the pathology of α-syn in neurodegenerative diseases, and further studies to address the key pathology of α-syn in the pathogenesis of FCD IIb, and TSC might benefit and have the potential to alter the progression of FCD IIb and TSC.

## 2. Materials and methods

### 2.1. Human brain tissue specimens

This study was conducted in accordance with the Declaration of Helsinki and the guidelines for the conduction of research involving human subjects, as established by the Ethics Committee of Xinqiao Hospital, Army Medical University. FCD IIb and TSC-induced pediatric drug-resistant epilepsy were diagnosed according to the current international consensus classification proposed by the ILAE. Invasive depth electrode recordings were performed if the epileptic lesions could not be precisely located by scalp electroencephalogram before the surgery. Only epileptogenic tissues that were identified with the dysplastic cortex by magnetic resonance imaging and further confirmed *post-hoc* by neuropathological correction were used in the study. Informed written consent was obtained from all participants or legal guardians for the use of the dissected tissue for research purposes only.

In the present study, 15 FCD IIb and 24 TSC lesions were examined. The general clinical information of all the patients is summarized in Table 1, and detailed clinical information of each patient is presented in Supplementary Tables S1, S2. Among the 26 control cortex (CTX) used in this study, 23 control tissues were obtained from patients with craniocerebral tumors in accordance with the criteria reported in previous studies during surgery (31, 32). None of any other neurological diseases or history of seizures were detected in these patients, and only the normal-appearing tissues without reactive astrogliosis, inflammation, or necrosis were included in the study. The other three control cortical specimens were obtained from individuals who were diagnosed with increased intracranial pressure due to traumatic brain injury and without any neurological disease in history. The samples distant from the directly injured area were obtained in <6h after brain injury (33) after comprehensive presurgical assessment, including history, neuroimaging studies, and neurological examination according to the criteria (27, 34). The detailed information of each control subject is shown in Supplementary Table S3.

**Table 1:** Summary of the clinical characteristics of patients with FCD IIb and TSC lesions.

	**FCD IIb**	**TSC**
Male/female	10/5	14/10
Median age of surgery (years)	13.3(3.5–34.0)	8.9(2.0–32.0)
Median age at onset of seizure (years)	5.1(0.2–18.0)	3.0(0.3–14.8)
Seizure type	FAS (1/6.7%) FIAS (1/6.7%) FBTCS (2/13.3%) GAS (1/6.7%) GTS (3/20%) GTCS (7/46.7%)	FAS (3/12.5%) FIAS (5/20.83%) FBTCS (1/4.1%) GAS (2/8.3%) GTCS (13/54.17%)
Lesion location	Frontal (9/60%) Temporal (5/33.3%) Parietal (1/6.7%)	Frontal (15/62.5%) Temporal (4/16.7%) Parietal (3/12.5%) Occipital (2/8.3%)
Median duration of epilepsy (years)	4.3 (0.9–20.0)	2.50 (0.2–30.0)
Median seizure frequency (per month)	105.0 (1–3000)	12.5 (1–300)
Postoperative outcome (Engle's class)	I (13/86.7%) III (1/6.7%) IV (1/6.7%)	I (18/75%) II (2/8.3%) III (2/8.3%) IV (2/8.3%)

### 2.2. Animal experiments

Sprague–Dawley rats were obtained from Army Medical University and housed under standard conditions with room temperature of 23 ± 1°C, humidity of 60%, and 12-h light/12-h dark cycle with food and water *ad libitum*. Randomly chosen female rats were exposed to X-ray (145 cGy, 60 s) (RS-2000, Rad Source Technologies) at post-pregnancy day 17 to disrupt the normal development of cortex *in utero*, and newborn rats were weaned at postnatal day 21 (P21) after normal delivery according to previous procedures (9). A total of 58 rats (both male and female) were used in this study, including 16 rats at 7 days: male/female: 7/9, 22 rats at 14 days: male/female: 12/10, and 20 rats at 28 days: male/female: 10/10. The number ratio of male to female was close to 1:1.

All experimental procedures were conducted in accordance with the guidelines of the Ethical Committee of Army Medical University and approved by the Internal Animal Care and Use Committee of Army Medical University.

### 2.3. RT-PCR

The total RNA was extracted from each specimen using the TRIzol reagent following the manufacture protocol (Invitrogen, Carlsbad, CA). RNA concentration was determined using a nanodrop spectrophotometer (Ocean Optics, Dunedin, FL) at 260/280 nm with a ratio of 1.8–2.0. Single-stranded cDNA was reverse-transcribed from 1 μg RNA using oligo dT primers (Takara, Otsu, Japan). The primers used in this study were as follows: human α*-syn* (forward: 5'GACAGCAGTAGCCCAGAAGACAG3', reverse: 5'GCATTTCATAAGCCTCATTGTCAG3'); human *GAPDH* (forward: 5'CACTCCTCCACCTTTGACGC3', reverse: 5'CTGTTGCTGTAGCCAAATTCGT3'), rat *syn* (forward: 5'GTTTTGTCAAGAAGGACCAGATG3', reverse: 5'GGCATTTCATAAGCCTCACTG3'); rat *GAPDH* (forward: 5'AAGTTCAACGGCACAGTCAAGG3', reverse: 5'CGCCAGTAGACTCCACGACATA3').

The PCR cycle was set as follows: 95°C for 30 s (one cycle), and 95°C for 5 s and 60°C for 40 s (49 cycles) to denaturation, 65°C for 5 s to anneal, and 95°C for 5s to extension. Each quantitative PCR reaction was repeated three times, and the quantitative PCR analysis was repeated by at least two independent experiments. The relative expression of α-syn mRNA in each sample was determined by the 2^−ΔΔCt^ method with GAPDA as internal control.

### 2.4. Western blotting

Protein of each specimen was extracted by the homogenizing tissue in RIPA lysis buffer supplemented with a mixture of protease and phosphatase inhibitors (cat.no: P0013B, Beyotime Biotechnology), and the concentration was determined using the Bradford protein assay (cat.no: P0010, Beyotime Biotechnology). An equal amount of protein (40 mg/ lane) was separated on 12% SDS-polyacrylamide gels and transferred to polyvinylidene fluoride membranes with a pore size of 0.2 μm. Membranes were blocked in 5% non-fat milk for 1 h and incubated with primary antibody overnight at 4°C (α-syn: 1:500, cat.no: 10842–1-AP, Proteintech; GAPDH:1:5000, cat.no: HRP-60004, Proteintech). Membranes were washed three times with Tris-buffered saline containing 0.1% Tween-20 (TBST) and then incubated with a peroxidase-conjugated goat anti-rabbit secondary antibody (1:1.000, cat.no: SA00001–2, Proteintech) for 1 h at 37°C. The signal was detected using chemiluminescent substrates (cat.no: SQ201L, Epizyme Biomedical Technology) after washing three times with TBST. GAPDH was used as the loading control. Densitometric analysis was performed using ImageJ to quantify the optical density (OD value) of each protein band.

### 2.5. Immunostaining

Paraffin-embedded tissues were sectioned at a thickness of 4 μm and mounted on polylysine-coated slides. Antigen retrieval was performed by heating sections in citrate buffer solution (0.01 M, pH 6.0) at 100°C for 30 min after deparaffinization and rehydration. After cooling to room temperature, the sections were washed with PBS. Sections were treated with 0.3% H_2_O_2_ to remove endogenous peroxidase activity. After blocked with 0.3% H_2_O_2_ for 10 min at 37°C, sections were incubated with primary antibodies: α-syn (1:200, cat.no:10842–1-AP, Proteintech, Wuhan), p-α-syn antibody (1:200, cat.no: PA5118553, Thermo Fisher) or NMDAR2A (1:100, cat.no: #M264, Sigma-Aldrich), or NMDAR2B (1:100, cat.no: 21920-1-AP, Proteintech) overnight at 4°C. Sections were incubated with HRP-conjugated secondary antibody (ZLI-9018, ZSBIO) for 1 h at 37°C and developed with the DAB Chromogenic Kit (ZLI-9018, ZSBIO) after washing three times with PBS. Counterstaining was performed with hematoxylin. For hematoxylin and eosin (HE) staining, sections were incubated with hematoxylin for 5 min and eosin for 20 s consecutively after deparaffinization and rehydration. An inverted bright-field microscope (BX63, OLYPUS, Japan) was used to acquire images.

For immunofluorescent staining, paraffin-embedded sections were blocked in 10% goat serum after deparaffinization and antigen retrieval and incubated with corresponding primary antibodies: α-syn (1:200, cat.no: 10842-1-AP, Proteintech), p-α-syn antibody (1:200, cat.no: PA5118553, Thermo Fisher), vesicular glutamate transporters 2 VGLUT2 (1:200, cat.no: ab211869, Abcam), vesicular GABA transporters VGAT (1:50, cat.no: ab211534, Abcam), vimentin (1:500, cat.no: 60330-1-Ig, Proteintech), NeuN (1:5000, cat.no: ab104224, Abcam), GFAP (1:400, cat.no: C9205, Sigma-Aldrich), Iba1 (1:500, cat.no: OB-PGP049, Biofarm), and Olig2 (1:500, cat.no:OB-PGP040-02, Biofarm) overnight at 4°C. After washing three times with PBS, the sections were incubated with Alexa Fluor 488-conjugated anti-rabbit antibody (1:1000, cat.no: #A-11008, Thermo Fisher Scientific), Alexa Fluor 555-conjugated anti-rabbit antibody (1:1000, cat.no: #A-21428, Thermo Fisher Scientific), Alexa Fluor 555-conjugated anti-mouse antibody (1:1000, cat.no: #A-21422, Thermo Fisher Scientific), or Alexa Fluor 488-conjugated anti-Guinea pig antibody (1:1000, cat.no: ab150185, Abcam) for 1 h at 37°C. Sections were subsequentially nuclear stained by Hoechst 33258 and photographed by a confocal microscope (LSM880; ZEISS, Germany).

Notably, the distinct morphology of BCs and astrocytes helps to distinguish them in FCD samples. BCs have eccentric nuclei, a ballooned cytoplasm, and do not exhibit clear axonal or dendritic processes (35). However, astrocytes exhibit a morphology of several stem branches that give rise to many finely branching processes in a uniform distribution. Additionally, Vimentin is generally used as a marker of immature glia and neurons during development (36) and was also used as a maker for BCs traditionally, which were demonstrated to be immature neurons in FCD lesions (37–40). However, GFAP is commonly used as a mature astrocytic marker (41, 42) and rarely detected in BCs in FCD lesions (27, 43, 44).

### 2.6. Co-immunoprecipitation

In brief, proteins were extracted from cortical tissues of FCD patients and rats using the Universal Magnetic Co-IP Kit protocol (cat. no: 54002, Active motif) under manufacturer's instructions. Protein extracts were incubated with protein G PLUS-Agarose (Santa Cruz, Biotechnology) for 1 h at 4 °C to eliminate non-specific binding. After incubation, the precleared supernatants containing 1 mg of protein were incubated with corresponding primary antibodies: α-syn (cat.no: #4179S, cell signaling technology), NMDAR2A (cat.no: #M264, Sigma-Aldrich), NMDAR2B (cat.no: #14544, cell signaling technology), or IgG (cat.no: #5127, cell signaling technology) overnight at 4 °C. Lysates were incubated with protein G PLUS-Agarose for 3 h at 4 °C with rotation. Beads were washed four times with complete Co-IP/wash buffer. Pre-IP lysates and bound proteins eluted from the immune complexes were denatured by heating to 95 °C for 5 min and used for Western blot analysis (12% gel for α-syn, 6% gel for NMDAR2A and NMDAR2B).

### 2.7. Evaluation of IHC

The immunoreactivity of α-syn and p-α-syn in DNs, BCs, and GCs was evaluated following an evaluation scheme reported previously (45), and the relative number of positive cells within cortical lesions was semi-quantified according to a three-point frequency score: 1: 0–10% (rare); 2: 11–50% (sparse); 3: > 50% (high), and the staining intensity was semi-quantified to a 4-point scale: 0 (absence), 1 (weak), 2 (moderate), and 3 (strong). The product of these two scores (frequency and intensity) yielded the total score. All the areas of the slices were evaluated, and the score represents the predominant cell staining intensity in each section.

The immunoreactivity of α-syn in neuropils, p-α-syn, NMDAR2A, and NMDAR2B was evaluated with image J in consistent with our previous study (46). In short, non-overlapping fields (400 × magnification, 0.3481 mm × 0.2621 mm width) were defined in the center of the lesions. Three sections from each specimen were selected, and three visions were captured in each section, and the ODs of the nine areas were averaged for the specimen. The immunoreactivity was evaluated by two histologists who were blind to the clinical data.

### 2.8. Statistics

The proper sample size and study power were estimated according to the previously established experimental settings (47). Data acquisition and analysis were done blindly and were presented as mean ± SEM. For comparisons between two independent groups, the unpaired two-tailed *t*-test was used. Spearman's rank correlation test was used for bivariate correlation analyses. Statistical methods and the number of replicates were indicated when used. Normality and equal variance tests were performed for all statistical analyses. GraphPad Prism 7 software was used for data plotting and analysis. A *p*-value of < 0.05 was considered as statistically significant.

## 3. Results

### 3.1. The expression profiles of α-syn in lesions of patients with FCD IIb and TSC

**Figure 1:**
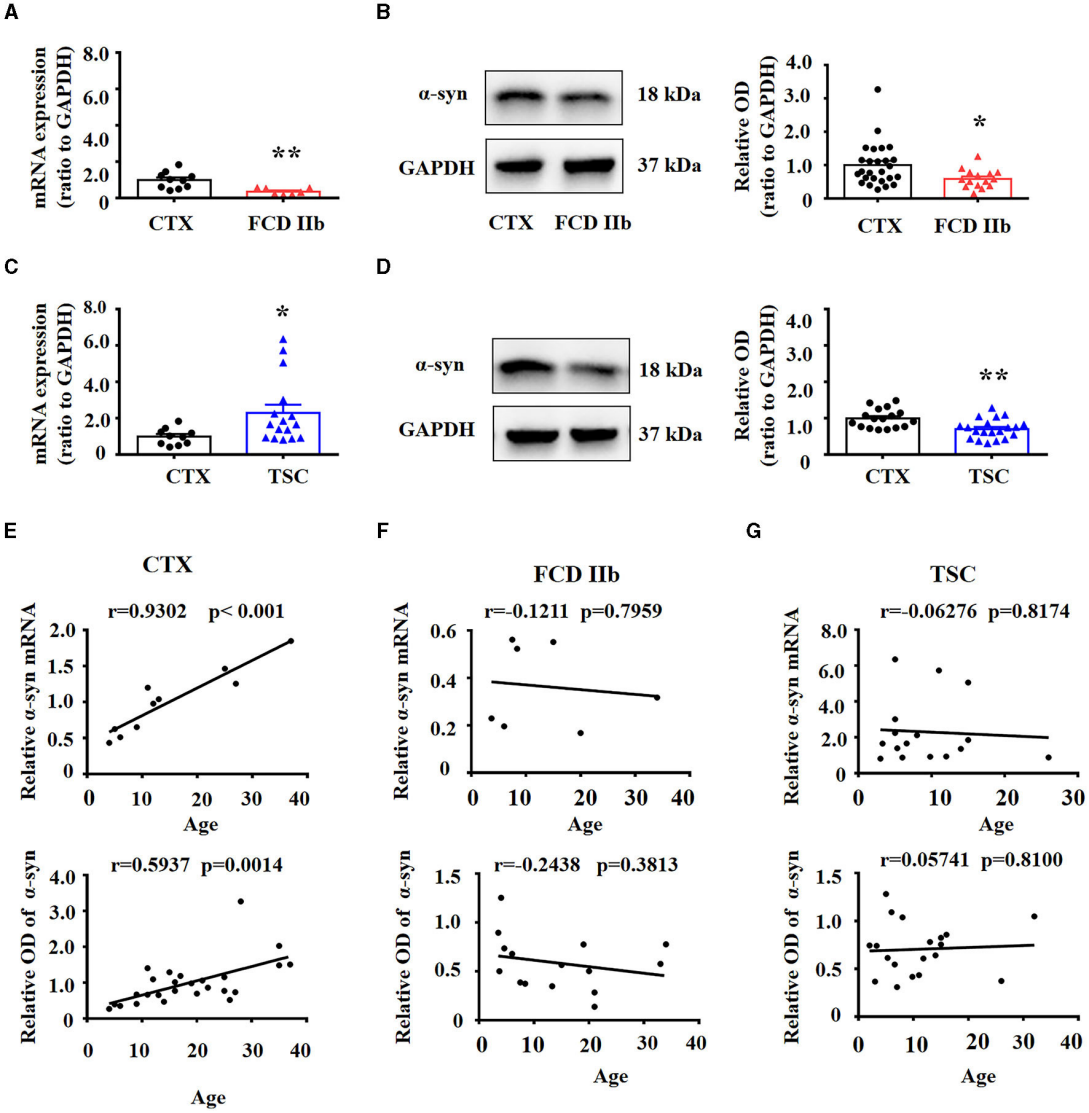
Expression of α-syn mRNA and protein in cortical lesions of patients with FCD IIb and TSC. **(A)** Statistical analysis showed that α-syn mRNA was significantly reduced in FCD IIb lesions (^**^p < 0.01, unpaired two-tailed *t*-test, *n* = 10, 7 specimens for each group). **(B)** Representative immunoblot bands showing α-syn was detected at approximately 18 kDa, and GAPDH (37 kDa) was used as the loading control. Densitometric analysis showed that α-syn protein was significantly reduced in FCD IIb lesions (^*^p < 0.05, unpaired two-tailed *t*-test, *n* = 26, 15 specimens for each group). **(C)** Statistical analysis showed that α-syn mRNA was increased in TSC lesions (^*^p < 0.05, unpaired two-tailed *t*-test, *n* = 10, 16 specimens for each group). **(D)** Immunoblot bands showing α-syn (18 kDa) and GAPDH (37 kDa) in total homogenates from CTX and TSC lesions. Densitometric analysis showed α-syn protein was significantly decreased in TSC lesions (^**^p < 0.01, unpaired two-tailed *t*-test, *n* = 17, 20 specimens for each group). **(E)** Spearman's correlation analysis showed that both α-syn mRNA (*r* = 0.9302, ^***^p < 0.001) and protein (*r* = 0.5937, *p* = 0.0014) were positively correlated with the ages of control subjects. **(F)** No significant correlation was observed between α-syn mRNA (*r* = −0.1211, *p* = 0.7959), protein (*r* = −0.2438, *p* = 0.3813), and ages of patients with FCD IIb. **(G)** No significant correlation was observed between α-syn mRNA (*r* = −0.06276, *p* = 0.8174), protein (*r* = 0.05741, *p* = 0.8100), and ages of patients with TSC.

We first examined the expression differences of α-syn between FCD IIb, TSC lesions, and control cortex (CTX) individually. RT-PCR results showed the expression of α-syn mRNA was significantly reduced in FCD IIb lesions (Figure 1A, ^**^p < 0.01, unpaired two-tailed *t*-test), α-syn band was detected at approximately 18 kDa by Western blotting, and quantitative analysis showed the expression of α-syn was significantly reduced in FCD IIb lesions (Figure 1B, ^*^p < 0.05, unpaired two-tailed *t*-test). Interestingly, RT-PCR results showed α-syn mRNA was significantly increased in TSC lesions (Figure 1C, ^*^p < 0.05, unpaired two-tailed *t*-test), while α-syn protein was reduced (Figure 1D, ^**^p < 0.01, unpaired two-tailed *t*-test). α-syn protein expression was increased gradually along with the development in the brain (48, 49). We found that both the mRNA and protein expression of α-syn were positively correlated with the ages of control subjects by Spearman's correlation analysis (Figure 1E). However, the positive correlation between α-syn and age was lost in patients with FCD IIb (Figure 1F) and TSC (Figure 1G).

### 3.2. Expression patterns of α-syn in FCD IIb and TSC lesions

**Figure 2:**
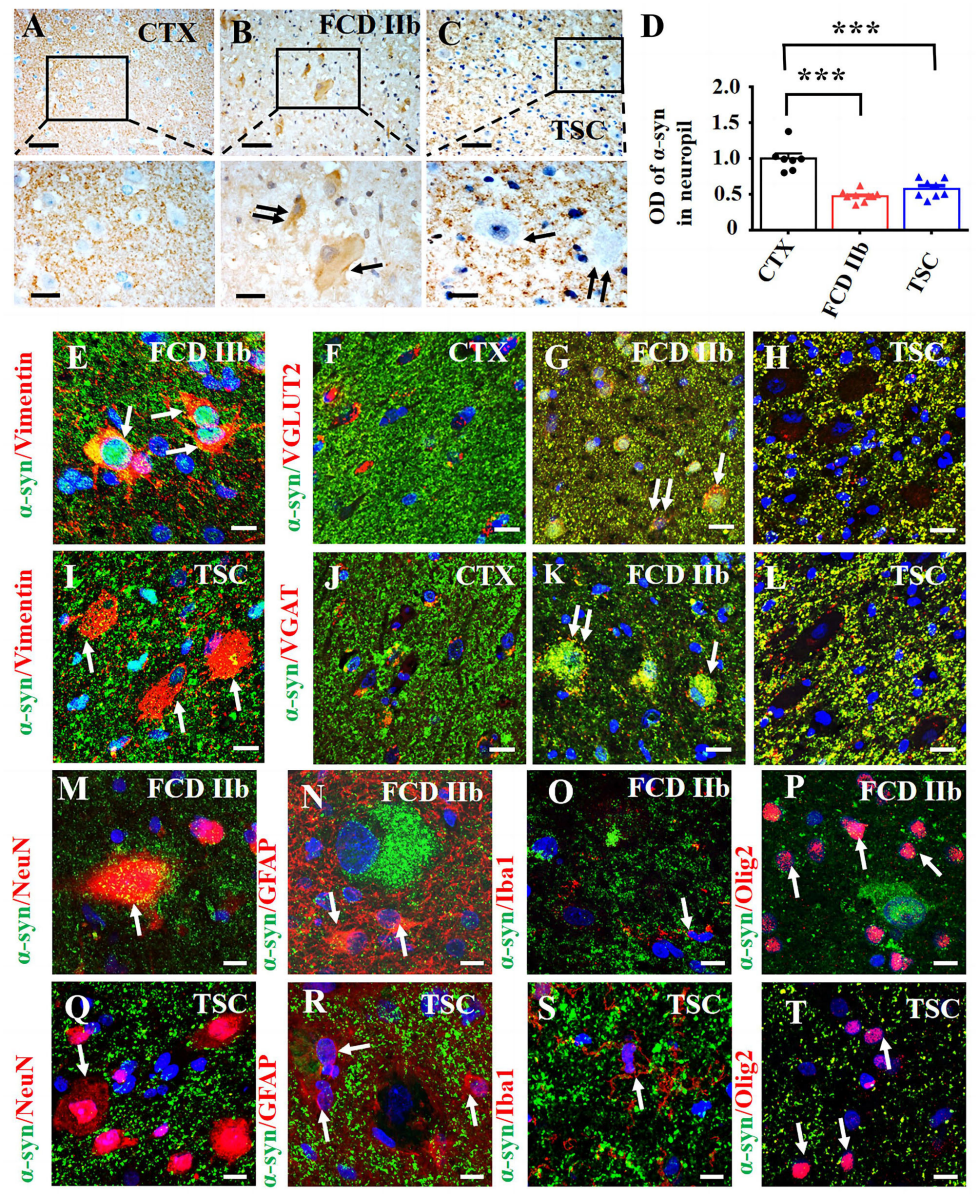
Expression profiles of α-syn in lesions of patients with FCD IIb and TSC. **(A)** Moderate-to-strong α-syn immunoreactivity was observed in the neuropils of CTX but was neglectable in the neuronal cytoplasm. **(B)** Representative image showing weak α-syn signal in the neuropils of FCD IIb lesions and moderate α-syn immunoreactivity in DN (double arrow) and BC (arrow) of FCD IIb lesions. **(C)** Strong but less of α-syn positive signal was observed in the neuropils of TSC lesions, and α-syn immunoreactivity was barely detected in DN (arrow) and GC (double arrow) of TSC lesions. **(D)** Statistical analysis results showing that the average OD value of α-syn was reduced in neuropils of both FCD IIb and TSC lesions (***p < 0.001, unpaired two-tailed *t*-test, *n* = 7, 9, 8 specimens for each group). **(E)** Representative images showing α-syn was expressed in vimentin-positive BC in FCD IIb lesions (arrows). **(F)** α-syn was barely expressed in VGLUT2-positive excitatory neurons in CTX. **(G)** Representative image showing the co-expression of α-syn and VGLUT2 was intensified in the neuropils, DN (double arrow), and BC (arrow) in FCD IIb lesions. **(H)** The co-expression of α-syn and VGLUT2 was intensified in the neuropils of TSC lesions as well but not observed in DN and BC. **(I)** α-syn was not detected in vimentin-positive GCs in TSC lesions. **(J)** Representative image showing α-syn was barely expressed in VGAT-positive neurons in CTX. **(K)** Representative image showing significant co-expression of α-syn and VGAT in DN (double arrow) and BC (arrow) of FCD IIb lesions. **(L)** The co-expression of α-syn and VGAT was intensified in the neuropils of TSC lesions but not observed in DN and GC of TSC lesions. **(M)** Representative image showing slightly α-syn expression in a NeuN-positive neuron and nearby neuropils in FCD IIb lesions (arrow). **(N–P)** Representative images showing α-syn was not detected in GFAP-positive astrocytes (**(N)**, arrows), Iba1-positive microglia (**O**, arrow), and Olig2-positive oligodendrocytes (**(P)**, arrows) in FCD IIb lesions. **(Q–T)** Representative images showing α-syn was not expressed in NeuN-positive neurons in TSC lesions (**(Q)**, arrow), GFAP-positive (**(R)**, arrows), Iba1-positive microglia (**(S)**, arrows), and Olig2-positive oligodendrocytes in TSC lesions (**(T)**, arrow). Scale bars: 50 μm for the upper panel of **(A–C)**, 20 μm for the bottom panel of **(A–C)**, and 10 μm for images **(E–T)**.

We investigated the cellular expression and distribution of α-syn in FCD IIb and TSC lesions with IHC. Moderate-to-strong immunoreactivity of α-syn was observed in the neuropils of CTX, while the immunoreactivity of α-syn was barely detected in the cytoplasm of neurons (Figure 2A). However, weak-to-moderate α-syn immunoreactivity was detected in DNs (double arrows) and BCs (arrows) in FCD IIb lesions (Figure 2B). Interestingly, α-syn immunoreactivity was barely observed in DNs (double arrows) in TSC lesions nor GCs (arrows) (Figure 2C). Additionally, weak α-syn immunoreactivity was observed in the neuropils of FCD IIb and TSC lesions (Figures 2B, C). Statistical analysis showed that the average optical density (OD) of α-syn was significantly reduced both in FCD IIb and TSC lesions (Figure 2D, ^***^p < 0.001, unpaired two-tailed *t*-test).

Additionally, we evaluated the immunoreactivity score of α-syn in the cytoplasm of control neurons, DNs, BCs, and GCs of FCD IIb and TSC lesions with methods reported in previous studies (35, 45) and summarized the information in Table 2. Specifically, α-syn immunoreactivity was not observed in the cytoplasm of neurons in CTX and was scored zero, but it was significantly increased in DNs, BCs of FCD IIb lesions and scored 3.67 ± 0.44, 3.33 ± 0.33, respectively (^***^p < 0.001, unpaired two-tailed *t*-test), and α-syn was not observed in DNs and GCs of TSC lesions and scored zero individually.

**Table 2:** α-syn and p-α-syn immunoreactivity score in cortical lesions of patients with FCD IIb and TSC.

		**Control**	**FCD IIb**	**TSC**
α-syn	DNs	—	3.67 ± 0.44^***^	0 ± 0
	BCs/GCs	—	3.33 ± 0.33	0 ± 0
P-α-syn	DNS	2.86 ± 0.13	6.88 ± 0.67^***^	8.14 ± 0.86^***^
	BCs/GCs	—	7.86 ± 0.56	5.43 ± 1.09

*** p < 0.001, FCD IIb, TSC vs. CTX, respectively. Unpaired two-tailed t-test, n = 7–9 specimens for each group.

Double immunofluorescent staining results showed that α-syn was expressed in vimentin-positive BCs in FCD IIb lesions (arrows in Figure 2E). α-syn was intensively expressed in the neuropils of CTX and sparsely co-localized with VGLUT2, a marker of excitatory neurons in CTX (Figure 2F). However, the immunoreactivity of VGLUT2 was intensely increased in the neuropils of both FCD IIb and TSC lesions, as shown by the co-expression of α-syn and VGLUT2 (Figures 2G, H). Importantly, co-expression of α-syn and VGLUT2 was observed in DNs (double arrows) and BCs (arrows) in FCD IIb lesions (Figure 2G) but not in the GCs of TSC lesions (Figure 2H). In TSC lesions, α-syn signal was not detected in vimentin-positive GCs (Figure 2I) nor in VGAT-positive inhibitory neurons in the CTX (Figure 2J). However, the co-expression of α-syn and VGAT was intensified in DNs and BCs of FCD IIb lesions (Figure 2K) and neuropils of TSC lesions (Figure 2L). Thus, these results suggested that α-syn might contribute to regulating the excitatory and inhibitory synaptic transmission both in FCD IIb and TSC lesions.

α-syn was expressed both in the cytoplasm of NeuN-positive neurons and nearby neuropils (Figure 2M) but not in GFAP-positive astrocytes (Figure 2N), Iba1-positive microglia (Figure 2O), and Olig2-positive oligodendrocytes (Figure 2P) in FCD IIb lesions. Interestingly, α-syn was not expressed in NeuN-positive neurons (Figure 2Q) in TSC lesions, GFAP-positive astrocytes (Figure 2R), Iba1-positive microglia (Figure 2S), and Olig2-positive oligodendrocytes (Figure 2T).

### 3.3. Expression patterns of p-α-syn in FCD IIb and TSC lesions

p-α-syn, in which α-syn is phosphorylated at Ser129, is the major pathological form of α-syn. The aggregation of p-α-syn in the cytoplasm and nuclei of neurons was identified as pathological markers of Parkinson's disease and multiple system atrophy in the central nervous system, respectively (50). We evaluated the immunoreactivity score of p-α-syn in the DNs, BCs, and GCs of FCD IIb and TSC lesions as well. As illustrated in Table 2, weak p-α-syn immunoreactivity was observed in the neurons of CTX and scored 2.86 ± 0.13. However, the immunoreactivity score of p-α-syn was significantly increased in the DNs and BCs of FCD IIb lesions, which scored 6.88 ± 0.67 and 7.86 ± 0.56, respectively (^***^p < 0.001, unpaired two-tailed *t*-test), and DNs and GCs of TSC lesions, which scored 8.14 ± 0.86 and 5.43 ± 1.09, individually (^***^p < 0.001, unpaired two-tailed *t*-test).

**Figure 3:**
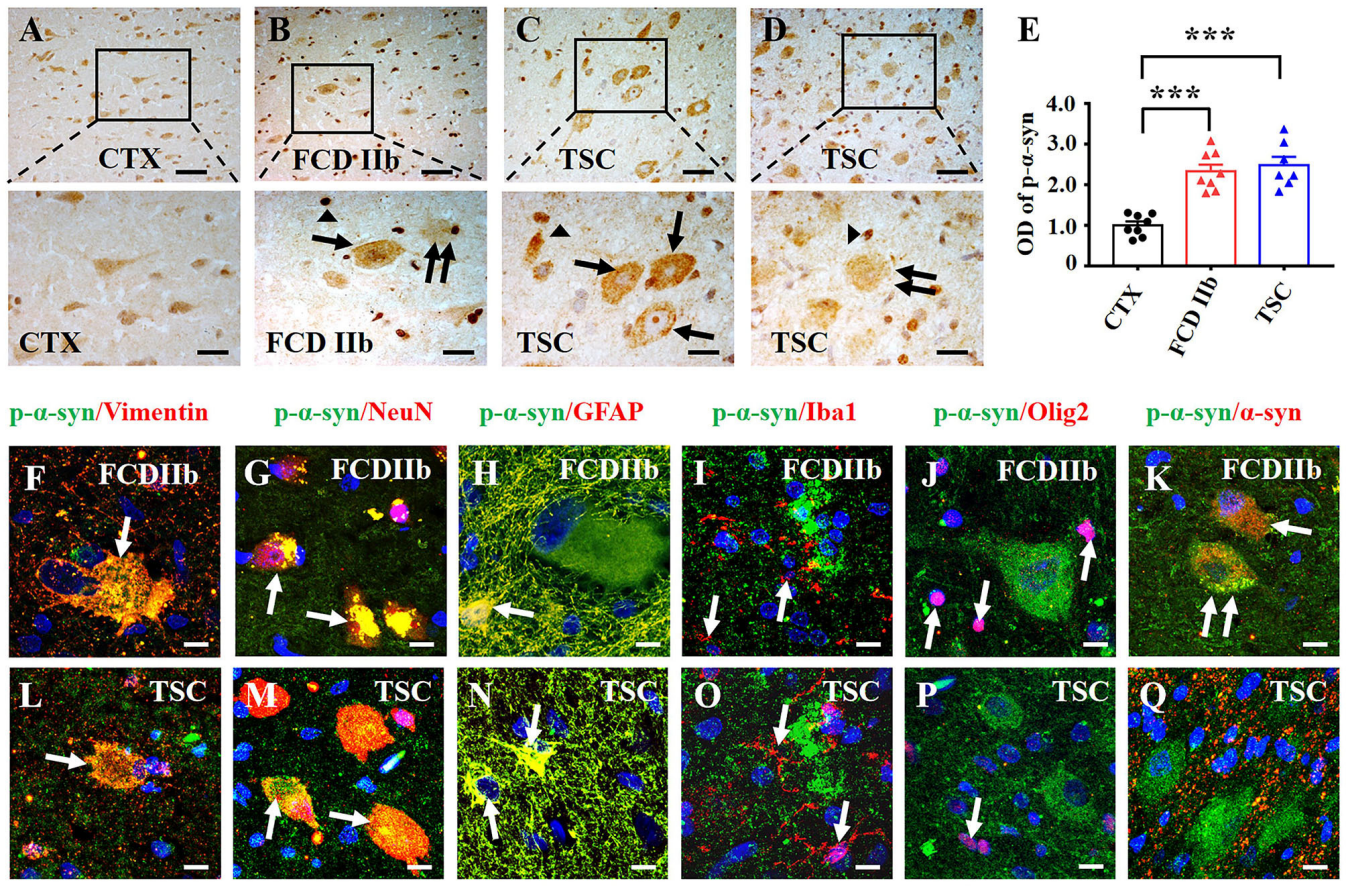
Expression profiles of p-α-syn in lesions of patients with FCD IIb and TSC. **(A)** Representative images showing moderate p-α-syn immunoreactivity in neurons of the CTX. **(B)** Moderate-to-strong p-α-syn immunoreactivity was observed in the cytoplasm of DN (arrow) and BC (double arrow) in FCD IIb lesions, and strong p-α-syn signal was detected in the nuclei of dysplastic neurons and glia-like cells (arrowhead). **(C, D)** Representative images showing strong p-α-syn immunoreactivity in DNs (arrow) and glia-like cells (arrowheads) of TSC lesions, and moderate p-α-syn immunoreactivity was observed in GC (double arrow). **(E)** Statistical analysis demonstrated that the average OD of p-α-syn was increased in cortical lesions of patients with FCD IIb and TSC (***p < 0.001, unpaired two-tailed *t*-test, *n* = 8, 8, 7 specimens for each group). **(F)** Representative image showing p-α-syn signal in vimentin-positive BC (arrow) in FCD IIb lesions. **(G, H)** Representative images showing p-α-syn immunoreactivity in NeuN-positive neurons (**G**, arrows) and GFAP-positive astrocytes in FCD IIb lesions (**H**, arrow). **(I, J)** Representative image showing p-α-syn was barely expressed in Iba1-positive microglia (**I**, arrows) and Olig2-positive oligodendrocytes (**J**, arrow). **(K)** Representative image showing p-α-syn and α-syn were co-expressed in in BC (arrow) and DN (double arrows) of FCD IIb lesions. **(L)** Representative image showing p-α-syn was expressed in the vimentin-positive GC in TSC lesions (arrow). **(M, N)** Representative images showing p-α-syn was detected in the NeuN-positive neurons (**M**, arrows) and GFAP-positive astrocytes in TSC lesions (**N**, arrow). **(O, P)** p-α-syn was barely observed in Iba1-positive microglia (**O**, arrow) and Olig2-positive oligodendrocytes (**P**, arrows) in TSC lesions. **(Q)** Representative images showing p-α-syn and α-syn were barely co-expressed in TSC lesions. Scale bars: 50 μm for upper panel of **(A–D)**, 20 μm for bottom panel of **(A–D)**, 10 μm for **(F–Q)**.

As shown by the representative images, weak-to-moderate immunoreactivity of p-α-syn was observed in neurons of CTX specimens (Figure 3A). In contrast, moderate-to-strong p-α-syn immunoreactivity was observed in DN (arrow), BC (double arrow), and glia-like cells (arrowheads) of FCD IIb lesions (Figure 3B). Strong p-α-syn immunoreactivity was detected in the cytoplasm and nuclei of DN (arrow) and glia-like cells (arrowheads) in TSC lesions (Figure 3C), while the intensity was relatively reduced in GC (double arrow) of TSC lesions (Figure 3D). Additionally, statistical analysis indicated the overall OD value of p-α-syn was increased in FCD IIb and TSC lesions (Figure 3E, ^***^p < 0.001, unpaired two-tailed *t*-test), suggesting that α-syn was abnormally aggregated in the dysplastic neurons in cortical lesions of patients with FCD IIb and TSC.

Double immunofluorescent staining results showed that p-α-syn was identified in vimentin-positive BC (Figure 3F) and NeuN-positive neurons in FCD IIb lesions (Figure 3G). Additionally, p-α-syn was detected in the GFAP-positive astrocytes (Figure 3H) but not in Iba1-positive microglia (Figure 3I) and Oligo2-positive oligodendrocytes (Figure 3J) in FCD IIb lesions. Furthermore, co-expression of α-syn and p-α-syn was observed in DN (arrow) and BC (double arrows) in FCD IIb lesions (Figure 3K). Intense p-α-syn signal was detected in vimentin-positive BC in TSC lesions (Figure 3L), NeuN-positive neurons (Figure 3M), and GFAP-positive astrocytes (Figure 3N). However, p-α-syn signal was not detected in Iba1-positive microglia (Figure 3O) and Olig2-positive oligodendrocytes (Figure 3P) in TSC lesions. Additionally, p-α-syn was barely co-expressed with α-syn in TSC lesions (Figure 3Q).

### 3.4. α-syn interacted with NMDAR in the cortical lesions of patients with FCD IIb and TSC

N-methyl-D-aspartic acid receptor (NMDAR) plays important role in mediating glutamate-induced excitatory synaptic transmission in the central nervous system (51). The molecular composition of NMDAR was found to be altered in the cortical samples of patients with FCD and relevant animal models (31, 52–54). Studies have shown that α-syn could alter synaptic transmission and plasticity by interfering with different NMDAR subunits (55–58). Therefore, we wondered whether α-syn regulates the expression of primary NMDAR subunits NMDAR2A and NMDAR2B in the cortical lesions of patient with FCD IIb and TSC and of FCD rats.

**Figure 4:**
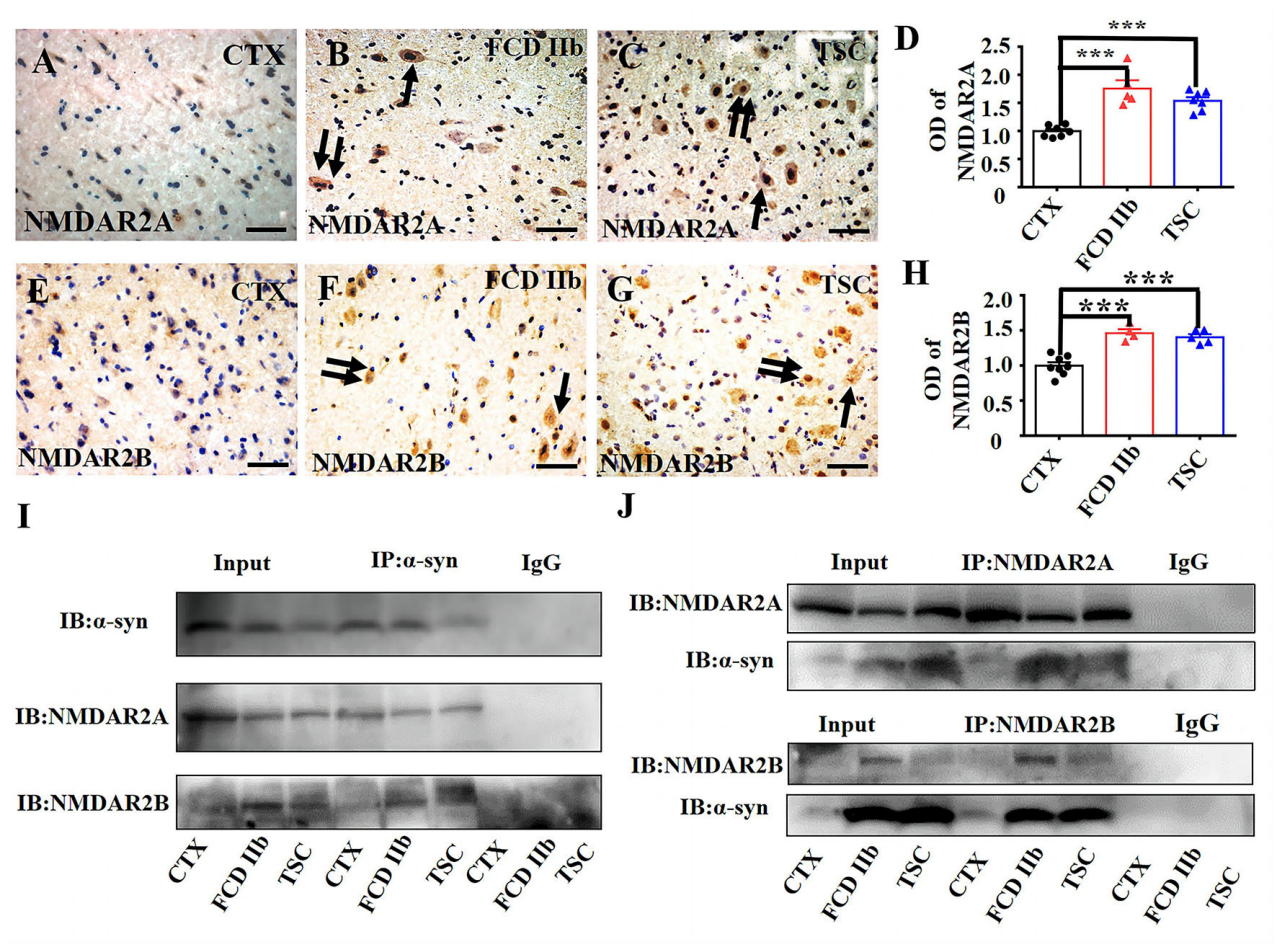
α-syn interacted with NMDAR in cortical lesions of patients with FCD IIb and TSC. **(A)** Weak NMDAR2A immunoreactivity was detected in the neurons in CTX. **(B, C)** Representative images showing strong NMDAR2A immunoreactivity in DNs (arrow) and BCs (double arrows) of FCD IIb lesions and also DNs (arrow) and BCs (double arrows) of TSC lesions. **(D)** Statistical analysis showed that the average OD of NMDAR2A was increased both in the FCD IIb and TSC lesions (^***^p < 0.001, unpaired two-tailed *t*-test, *n* = 7, 5, 7 specimens for each group). **(E)** Representative image showing weak NMDAR2B immunoreactivity in neurons of CTX. **(F, G)** Representative images showing strong NMDAR2B immunoreactivity in DNs (arrow) and BCs (double arrows) of FCD IIb lesions, and DNs (arrow) and GCs (double arrows) of TSC lesions. **(H)** Statistical analysis showed that the average OD of NMDAR2B was increased both in the FCD IIb and TSC leisons (^***^p < 0.001, unpaired two tailed *t*-test, *n* = 8, 4, 5 specimens for each group). **(I)** Representative immunoblotting bands showing that α-syn/NMDAR2A complex was not significantly altered, while α-syn/NMDAR2B complex was augmented both in FCD IIb and TSC lesions. IgG was used as negative control. **(J)** Representative immunoblotting bands showing significant augmentation of both NMDAR2A/α-syn and NMDAR2B/α-syn complex in FCD IIb and TSC lesions. IgG was used as negative control.

First, we investigated the expression profiles of NMDAR2A and NMDAR2B in cortical lesions of patients with FCD IIb and TSC. As shown by the representative images (Figures 4A–C, E–G), in comparison with the immunoreactivity of NMDAR2A and NMDAR2B in CTX, NMDAR2A and NMDAR2B immunoreactivity were intensively increased in the dysplastic neurons of FCD IIb and TSC lesions. Statistical analysis results showed that the immunoreactivity of both NMDAR2A and NMDAR2B was significantly increased in FCD IIb and TSC lesions (Figures 4D, H). Furthermore, we verified the interaction of α-syn with NMDAR2A and NMDAR2B in cortical lesions of patients with FCD IIb and TSC with Co-IP. Representative images and immunoblotting bands (Figure 4I) showed that the interaction of α-syn/NMDAR2A was not significantly altered, but the α-syn/NMDAR2B complex was augmented in FCD IIb and TSC lesions. However, reverse verification bands showed that the NMDAR2A/α-syn complex was significantly augmented both in FCD IIb and TSC lesions (Figure 4J, upper panel). In comparison with the blotting bands of NMDAR2A, the expression of NMDAR2B was relatively weak in the homogenates of CTX, FCD IIb, and TSC lesions (Figure 4J, bottom panel). However, the NMDAR2B/α-syn complex was significantly strengthened both in FCD IIb and TSC lesions, suggesting strong interactions of α-syn with NMDAR2A and NMDAR2B in the cortical lesions of patients with FCD IIb and TSC.

### 3.5. The expression profiles of α-syn and p-α-syn in FCD rats

**Figure 5:**
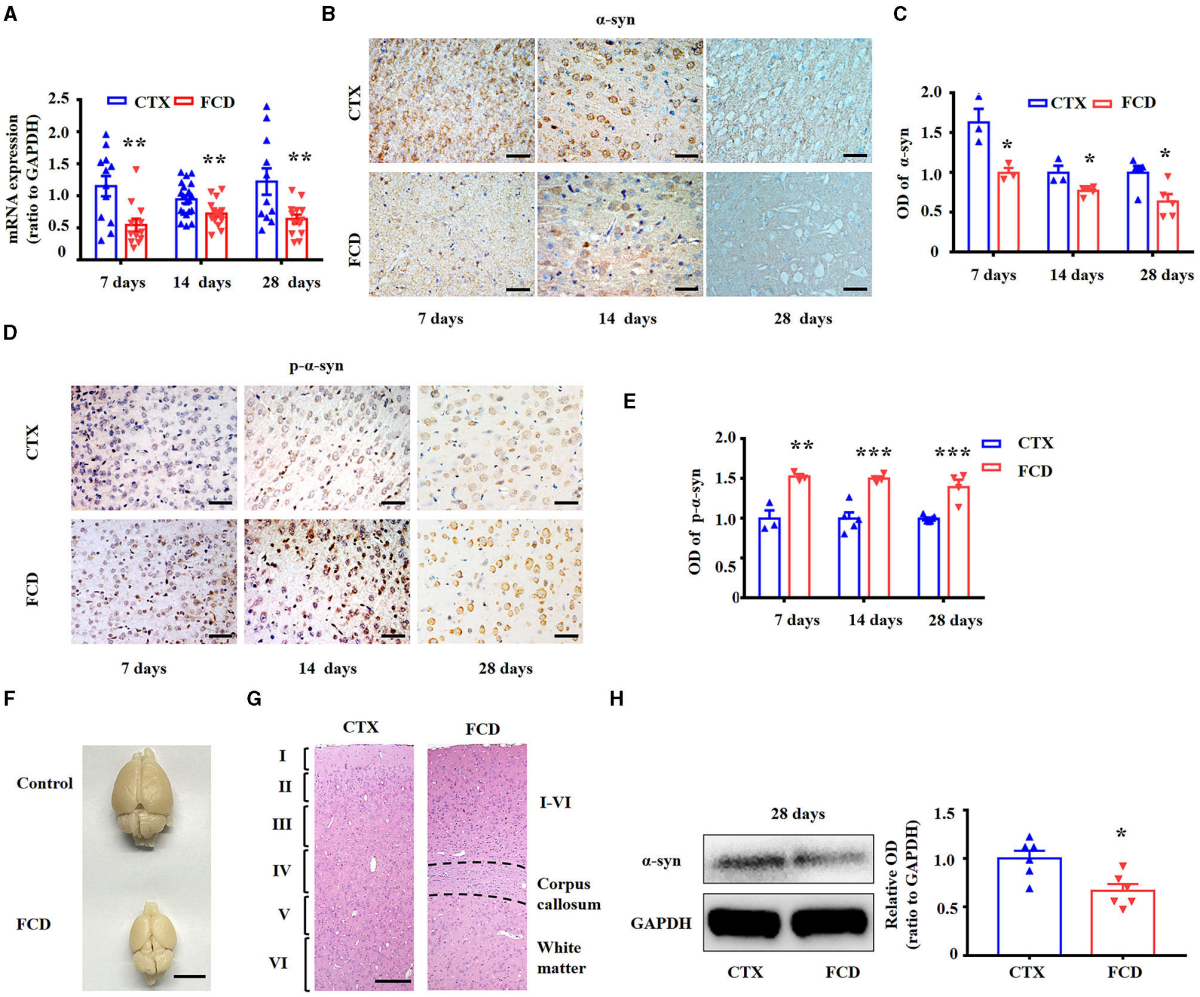
Expression profiles of α-syn and p-α-syn in FCD rats. **(A)** RT-PCR results showed α-syn mRNA expression was significantly reduced in FCD rats at postnatal 7, 14, and 28 days (**p < 0.01, unpaired two-tailed *t*-test, *n* = 11–20 rats for each group). **(B)** Representative images showing strong α-syn immunoreactivity in the neuronal membrane and cytoplasm or neuropils in the cortex of control rats but weak-to-moderate α-syn immunoreactivity in the cortex of FCD rats at 7, 14, and 28 days after birth. Scale bars: 50 μm. **(C)** Statistical analysis showed that the α-syn protein was significantly decreased in FCD rats (*p* < 0.05, unpaired two-tailed *t*-test, *n* = 3–5 rats for each group). **(D)** Weak p-α-syn immunoreactivity was observed in the neuronal membrane of control rats, whereas it was strengthened in the cortical neurons of FCD rats at 7, 14, and 28 days after birth. Scale bars: 50 μm. **(E)** Statistical analysis showed that the p-α-syn protein was significantly increased in FCD rats (**p < 0.01, ***p < 0.001, unpaired two-tailed *t*-test, *n* = 3–7 rats for each group). **(F)** Representative images showing deformed brain in FCD rats at 28 days after birth, as shown by reduced telencephalon and cerebellum volume, and uncovered quadrigemina. Scale bar: 1 cm. **(G)** HE staining images showing thinner cortex (indicated by the occurrence of corpus callosum) and disrupted lamellar structure in FCD rats at 28 days after birth. Scale bar: 200 μm. **(H)** Representative immunoblot bands of α-syn and GAPDH in total homogenates from the cortex of control and FCD rats at 28 days after birth, and statistical analysis results showing that α-syn protein was significantly decreased in FCD rats (*p < 0.05, unpaired two-tailed *t*-test, *n* = 6 rats for each group).

Consistent with previous studies (9, 27), we utilized *in utero* X-ray radiated rats to replicate the pathological characteristics of FCD. We used RT-PCR to determine α-syn mRNA expression in the FCD rats first. Statistical analysis showed that α-syn mRNA was significantly reduced in FCD rats from 7, 14, to 28 days after birth (Figure 5A, ^**^p < 0.01, unpaired two-tailed *t*-test). Pyramidal neurons in layer V are the principal output neurons in the cortex (59, 60). Thus, we explored the expression profiles of α-syn and p-α-syn in layer V of cortical lesions. IHC results showed that α-syn immunoreactivity was significantly reduced in layer V of cortical lesions in FCD rats from 7, 14, to 28 days after birth (Figures 5B, C). Conversely, IHC results showed that p-α-syn immunoreactivity was significantly increased in layer V of cortical lesions in FCD rats from 7, 14, to 28 days after birth (Figures 5D, E). As shown by the representative images, the appearance of the brain of X-ray-radiated rats (FCD rats) at 28 days after birth was dramatically deformed, illustrated by an apparent reduction in bulk volume, telencephalon, and cerebellum (Figure 5F). Furthermore, HE-staining results showed that the cortical structure was significantly disrupted, as shown by a much thinner cortex (indicated by the occurrence of corpus callosum) and non-obvious lamellar structure (Figure 5G), which was highly similar to the pathological characteristics of cortical dyslamination in FCD patients. Additionally, Western blotting results showed that α-syn protein expression was significantly reduced in FCD rats at 28 days after birth (Figure 5H).

### 3.6. α-syn interacted with NMDAR in the cortex of FCD rats

**Figure 6:**
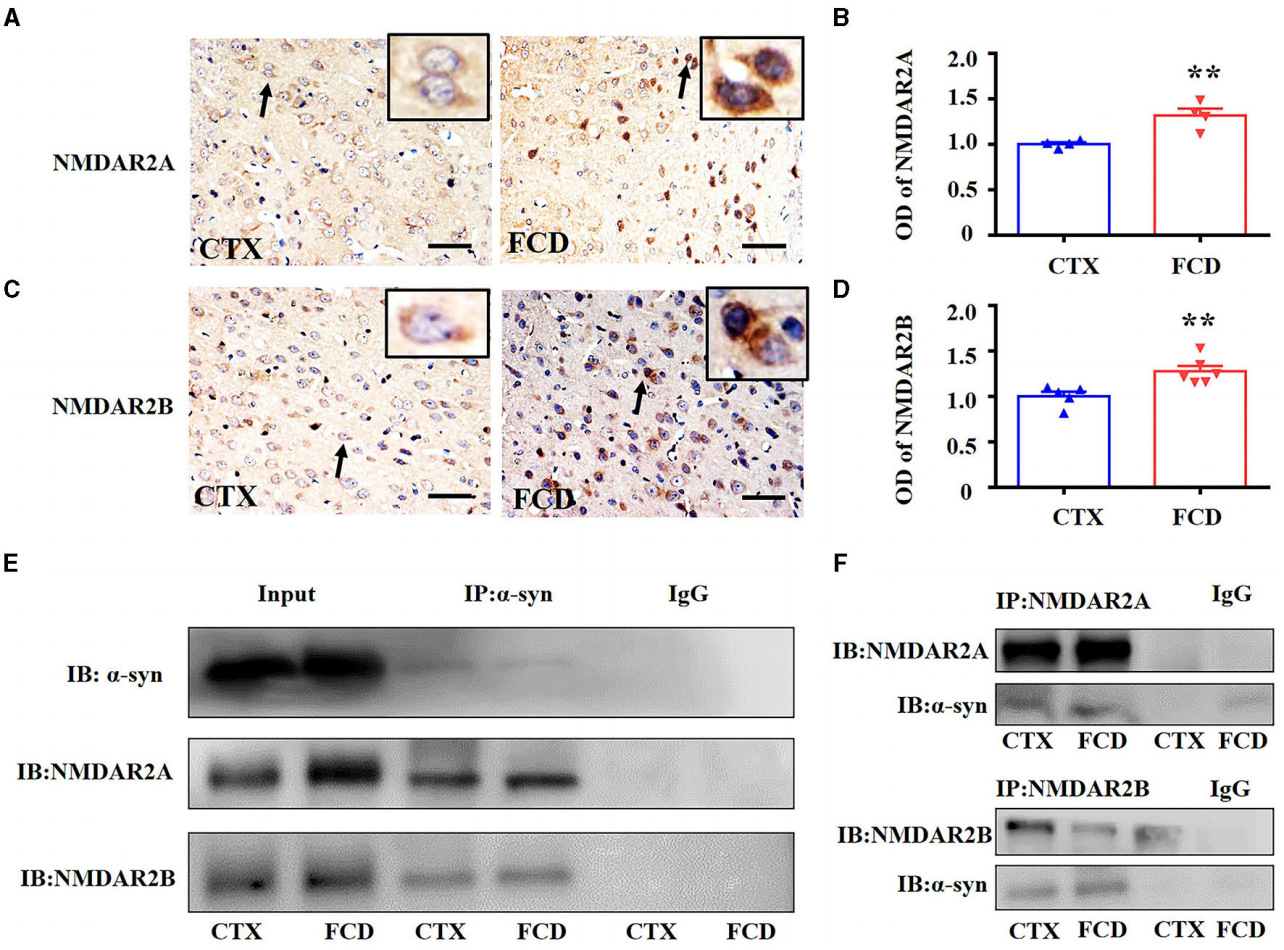
α-syn interacted with NMDAR in the cortex of FCD rats at postnatal 28 days. **(A)** Representative images showing moderate NMDAR2A immunoreactivity on layer V cortical neuronal membrane of control rats (arrow and inset) but was intensified both in the membrane and cytoplasm of cortical neurons in FCD rats (arrow and inset). **(B)** Statistical analysis showing NMDAR2A immunoreactivity was increased in the cortex of FCD rats (**p < 0.01, unpaired two-tailed *t*-test, *n* = 4 rats for each group). **(C)** Weak NMDAR2B immunoreactivity was observed in the cytoplasm of cortical neurons in control rats (arrow and inset) but increased immunoreactivity in the cortical neurons of FCD rats (arrow and inset). **(D)** Statistical analysis showed NMDAR2B immunoreactivity was significantly increased in the cortex of FCD rats (**p < 0.01, unpaired two-tailed *t*-test, *n* = 5, 6 rats for each group). **(E)** Representative Western blotting bands showing immunoprecipitation of α-syn in the cortex of control and FCD rats. Membranes were immunoblotted with anti-NMDAR2A and anti-NMDAR2B antibodies. Augmented α-syn/NMDAR2A and α-syn/NMDAR2B complex was observed in the cortical homogenates of FCD rats. IgG was used as negative control. **(F)** Representative Western blotting bands showing immunoprecipitation of NMDAR2A or NMDAR2B, membranes were immunoblotted with anti-α-syn antibody, and the augmentation of NMDAR2B/α-syn was more apparent in FCD rats. IgG was used as negative control.

Consistent with the experiments performed in FCD IIb and TSC patients, we investigated the expression of NMDAR2A and NMDAR2B in cortical neurons of layer V in FCD rats. IHC results showed that both the immunoreactivity of NMDAR2A and NMDAR2B were increased in the cortical neurons of FCD rats, as shown by the representative images and statistical results (Figures 6A–D) (^**^p < 0.01, unpaired two-tailed *t*-test.). To test whether α-syn interacts with NMDAR2A and NMDAR2B, we performed Co-IP to investigate the interaction between α-syn and NMDAR2A and NMDAR2B. As shown by the representative immunoblotting bands (Figure 6E), this qualitative approach suggested an augmentation in the levels of the α-syn/NMDAR2A and α-syn /NMDAR2B complex in FCD rats at 28 days after birth, and IgG was used as negative control. Reverse Co-IP was performed to confirm the interaction of NMDAR2A and NMDAR2B with α-syn. As shown by the representative immunoblotting bands (Figure 6F), the NMDAR2A/α-syn complex did not significantly differ between control and FCD rats by pulling down NMDAR2A, whereas the NMDAR2B/α-syn complex was intensified in FCD rats at 28 days after birth.

We also investigated the interaction of α-syn and NMDAR in FCD and control rats at postnatal 7 days and 14 days. At 7 days after birth, as shown in representative immunoblotting bands (Supplementary Figure S1A), α-syn/NMDAR2A complex and α-syn/NMDAR2B complex were reduced in the FCD rats. Reverse immunoblotting bands (Supplementary Figure S1B) showed that the NMDAR2A/α-syn complex did not significantly differ between the control and FCD rats by pulling down NMDAR2A, while the NMDAR2B/α-syn complex was augmented. At 14 days after birth, the immunoblotting bands (Supplementary Figure S1C) indicated that both α-syn/NMDAR2A complex and α-syn/NMDAR2B complex were intensified in the FCD rats, while reverse Co-IP immunoblotting bands (Supplementary Figure S1D) showed that NMDAR2A/α-syn and NMDAR2B/α-syn complex were reduced in the FCD rats. Together, these results suggested that α-syn might contribute to regulating the synaptic transmission in FCD rats by interfering with NMDAR from an early developmental stage.

## 4. Discussion

The present study highlighted the distinctive expression profiles of α-syn and p-α-syn, and the interaction of α-syn with NMDAR in the cortical lesions of patients with FCD IIb and TSC, and FCD rats generated by X-ray-radiation, suggesting a potential role of α-syn/NMDAR complex in the pathogenesis and epileptogenesis of FCD IIb and TSC.

α-syn is shown to be concentrated in nerve terminals in close proximity to synaptic vesicles, with little staining in somata and dendrites, implicated in crucial roles for in vesicular trafficking, neurotransmitter release, and neurotransmission (61, 62). Here, we found the expression of α-syn protein was significantly reduced in the cortical lesions of patients with FCD IIb and TSC. The inconsistency of increased mRNA level but reduced protein expression of α-syn in TSC patients was intriguing, and we speculated that mechanisms concerning posttranscriptional translation contribute to the discrepancy (63–65). However, the co-expression of α-syn with both VGLUT2 and VGAT was intensified in cortical lesions of patients with FCD IIb and TSC, suggesting a potential role of α-syn in regulating synaptic transmission during epileptic activity in patients with FCD IIb and TSC.

Fibrillary aggregates of α-syn protein, p-α-syn in the cytoplasm of neurons and glia termed as synucleinopathies, have been implicated in several neurodegenerative diseases, including Parkinson's disease, Alzheimer's disease, and multiple sclerosis atrophy (66). Studies reported that α-syn protein aggregation, including oligomeric deposits, protofibrils deposits, and fibrils, can spread across neurons and glias similar to the pathological characteristics of prionic diseases (67–69). Here, we found that p-α-syn was specifically aggregated in the cytoplasm, nuclei of BCs and GCs, dysplastic neurons, and astrocytes in the cortical lesions of patients with FCD IIb and TSC as well and further verified the neuropathological characteristics of dysplastic neurons and BCs and GCs in FCD IIb and TSC. Conversely, α-syn was absent in DNs and GCs of TSC lesions. These phenomena can be explained by the following reasons. α-syn was synthesized in neuronal cytoplasm and transported to nerve terminals gradually for functioning during development (70, 71). However, DN and GCs are typical dysplastic neurons with eccentric nuclei, giant cytoplasm, and without clear axonal or dendritic processes, suggesting that critical cytological deficits existed in these cells, which might indicate the underlying deficiency of α-syn in DN and GCs. Interestingly, positive p-α-syn immunoreactivity was observed in the cytoplasm of DN and GCs, possibly attributing to the disturbed activity of various kinases and signaling cascades in TSC lesions after the abnormal activation of mTOR pathway (72).

Our previous studies have shown that NMDAR-mediated currents were significantly increased in the dysplastic neurons in FCD rats (27). Consistent with the functional augmentation of NMDAR, we found that the immunoreactivity of NMDAR2A and NMDAR2B was consistently increased in the cortical lesions of patients with FCD IIb and TSC and also FCD rats in this study. Several studies have pointed out that α-syn could interact with NMDAR to induce synaptic dysfunction in the central nervous system, leading to memory and cognitive impairments in animals (55–57, 73, 74). Additionally, α-syn was reported to modulate the NMDAR2B activation and internalization in cultured cortical neurons (75). Here, we found that the direct interaction of α-syn with NMDAR2A and NMDAR2B was significantly intensified in FCD rats since postnatal 7 days, which suggested that α-syn might contribute to the synaptic dysfunction in FCD lesions by modulating the internalization and activation of NMDAR. Thus, our study here suggested a causal relationship between disturbed α-syn expression and abnormal NMDAR function in the pathogenesis and epileptogenesis of FCD IIb and TSC.

## 5. Conclusion

Taking together, our study characterized the disturbed expression of α-syn and p-α-syn in FCD and TSC lesions and demonstrated a stronger interaction between α-syn and NMDAR in cortical lesions of patients with FCD IIb and TSC, and FCD rats, proposing a potential linkage between α-syn and NMDAR in the pathogenesis and epileptogenesis of FCD. These results advanced our understanding of the potential function of α-syn by mediating NMDAR during the pathological development of FCD IIb and TSC. Studies concerning the modulating effects of α-syn on seizure activity in future would expand our understanding of the pathogenesis of FCD IIb and TSC further.

## Data availability statement

The original contributions presented in the study are included in the article/Supplementary material, further inquiries can be directed to the corresponding authors.

## Ethics statement

The studies involving humans were approved by the Ethics Committee of Xinqiao Hospital, Army Medical University. The studies were conducted in accordance with the local legislation and institutional requirements. Written informed consent for participation in this study was provided by the participants' legal guardians/next of kin. The animal study was approved by the Ethical Committee of Army Medical University and approved by the Internal Animal Care and Use Committee of Army Medical University. The study was conducted in accordance with the local legislation and institutional requirements. Written informed consent was obtained from the individual(s), and minor(s)' legal guardian/next of kin, for the publication of any potentially identifiable images or data included in this article.

## Author contributions

LZ: Data curation, Writing—original draft. JH: Methodology, Resources, Writing—review & editing. LD: Methodology, Writing—review & editing. GZ: Methodology, Writing—review & editing. X-LY: Formal Analysis, Methodology, Writing—review & editing. ZH: Methodology, Software, Writing—review & editing. Y-HL: Conceptualization, Resources, Writing—review & editing. HY: Resources, Writing—review & editing. C-QZ: Funding acquisition, Investigation, Methodology, Project administration, Resources, Writing—review & editing. K-FS: Formal Analysis, Investigation, Methodology, Software, Supervision, Writing—review & editing. PL: Methodology, Supervision, Writing—review & editing.
