# Supplementary material for: Expression profiles of α-synuclein in cortical lesions of patients with FCD IIb and TSC, and FCD rats
Source: Front Neurol. 2023 Nov 7;14:1255097. doi: 10.3389/fneur.2023.1255097 (PMC10662349; doi:10.3389/fneur.2023.1255097)
Supplement: Supplementary file 1 [file Data_Sheet_1.pdf]

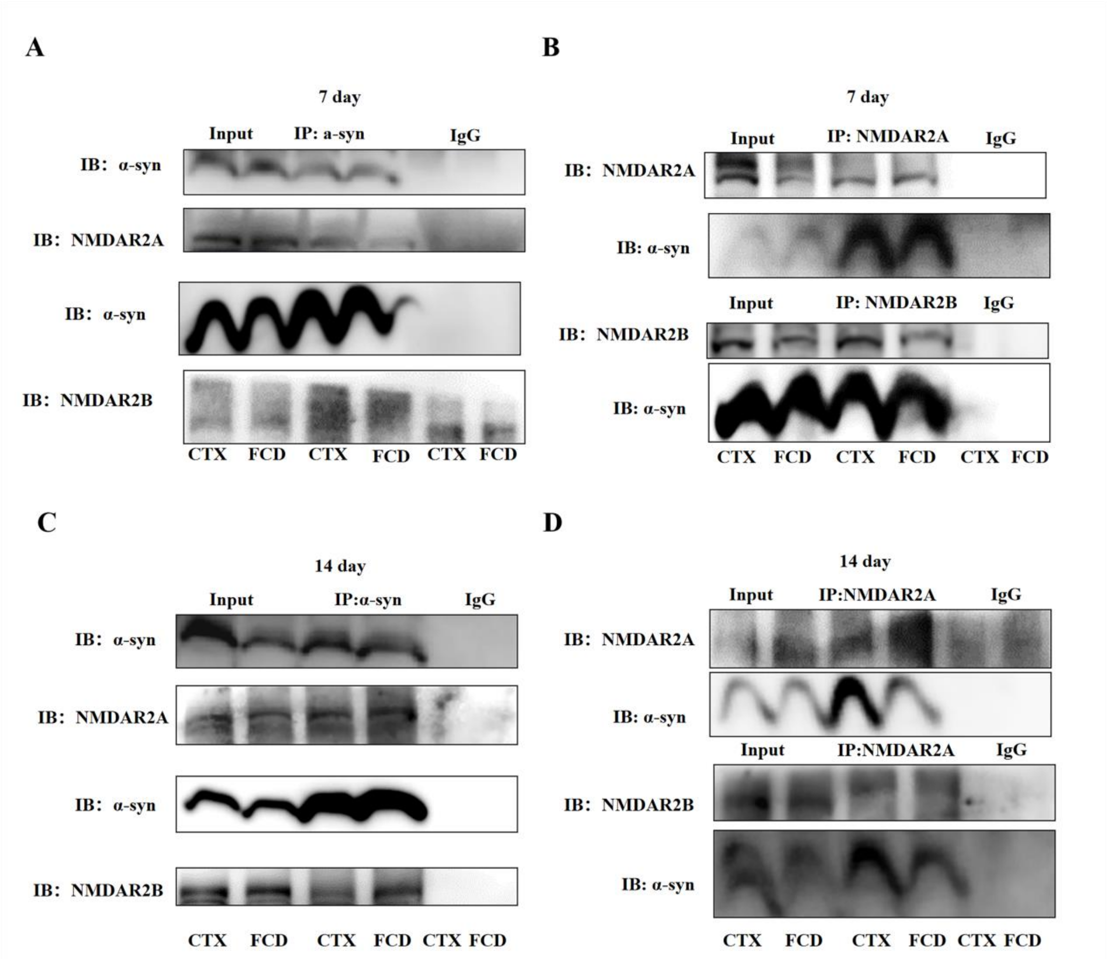


**Figure S1. Interaction between α-syn and NMDAR in cortical lesions of FCD rats at postnatal 7 and 14 days.**

(A) Representative western blotting bands showing immunoprecipitation of α-syn in the cortex of control, FCD rats at 7 days after birth. Membranes were immunoblotted with anti-NMDAR2A, anti- NMDAR2B antibodies. Decreased α-syn/NMDAR2A and α-syn/NMDAR2B complex were observed in the cortical homogenates of FCD rats at 7 days after birth. IgG was used as negative control. (B) Representative western blotting bands showing immunoprecipitation of NMDAR2A or NMDAR2B, membranes were immunoblotted with anti-α-syn antibody, the augmentation of NMDAR2B/α-syn was more obvious in FCD rats at postnatal 7 days. IgG was used as negative control. (C) Representative western blotting bands showing immunoprecipitation of α-syn in the cortex of control and FCD rats at postnatal 14 days. Membranes were immunoblotted with anti-NMDAR2A, anti- NMDAR2B antibodies. Increased α-syn/NMDAR2A and α-syn/NMDAR2B complex were

observed in the cortical homogenates of FCD rats at postnatal 14 days. IgG was used as negative control. (D) Representative western blotting bands showing immunoprecipitation of NMDAR2A or NMDAR2B, membranes were immunoblotted with anti-α-syn antibody, NMDAR2A/α-syn and NMDAR2B/α-syn complex were reduced in FCD rats at postnatal 14 days. IgG was used as negative control.

## Supplementary tables

Table1. Clinical and neuropathological features of patients with FCD IIb lesions.

| NO | Sex | Diseases | Age at surgery (Years) | Age of seizure (Years) | ED (Years) | Location | ST | SF (/Month) | ASMs | PO | Application | Relative α-syn mRNA | Relative OD of α-syn protein |
|---|---|---|---|---|---|---|---|---|---|---|---|---|---|
| 1 | F | FCD IIb | 3.5 | 0.2 | 3.3 | LF | GTCS | 105 | LEV<br>OXC | I | WB | | 0.90 |
| 2 | M | FCD IIb | 3.7 | 0.3 | 4.0 | LF | FAS | 3000 | LEV<br>OXC | I | RT-PCR<br>WB<br>IHC | 0.23 | 0.50 |
| 3 | M | FCD IIb | 4.0 | 1.0 | 3.0 | LF | FBTCS | 105 | LEV<br>LTG<br>VPA<br>TPM | I | WB<br>IHC | | 1.3 |
| 4 | M | FCD IIb | 4.6 | 0.3 | 4.3 | F | GTCS | 4 | VPA<br>LEA | I | WB | | 0.74 |
| 5 | M | FCD IIb | 6.0 | 5.1 | 0.9 | T | FBTCS | 600 | OXC<br>LEV<br>NZP | I | RT-PCR<br>WB<br>IHC | 0.20 | 0.68 |
| 6 | M | FCD IIb | 7.5 | 2.5 | 5.0 | RT | GTCS | 150 | VPA<br>OXC | I | RT-PCR<br>WB<br>IHC | 0.56 | 0.39 |
| 7 | F | FCD IIb | 8.4 | 6.4 | 2.0 | F | GTS | 120 | LEV<br>OXC | I | RT-PCR<br>WB<br>IHC | 0.52 | 0.37 |
| 8 | F | FCD IIb | 13.3 | 10.3 | 3.0 | RF | GTS | 75 | LEV<br>OXC | I | WB | | 0.35 |

| | | | | | | | | | | | | | |
|---|---|---|---|---|---|---|---|---|---|---|---|---|---|
| 9 | F | FCD IIb | 15.0 | 12.0 | 3.0 | LF | GTCS | 120 | OXC<br>NZP<br>LEV | I | RT-PCR<br>WB<br>IHC<br>IF | 0.55 | 0.57 |
| 10 | M | FCD IIb | 19.0 | 3.0 | 16.0 | LT | FIAS | 1 | VPA<br>OXC | III | WB | | 0.78 |
| 11 | M | FCD IIb | 20.0 | 7.0 | 13.0 | RF | GTCS | 150 | VPA<br>CBZ<br>PHT | I | RT-PCR<br>WB<br>IHC | 0.17 | 0.50 |
| 12 | M | FCD IIb | 21.0 | 9.0 | 12.0 | RT | GAS | 2 | VPA<br>CBZ<br>LTG | I | WB | | 0.14 |
| 13 | F | FCD IIb | 21.0 | 5.0 | 16.0 | RF | GTCS | 2 | VPA<br>LEA | IV | WB | | 0.28 |
| 14 | M | FCD IIb | 33.0 | 13.0 | 20.0 | P | GTS | 90 | CBZ<br>PB | I | WB<br>IHC<br>IF | | 0.58 |
| 15 | M | FCD IIb | 34.0 | 18.0 | 16.0 | LT | GTCS | 9 | TPM<br>CBZ | I | RT-PCR<br>WB<br>IHC | 0.32 | 0.78 |

M=Male; F=Female; ED: Epilepsy duration; ST: Seizure type; SF: Seizure frequency; ASMs: Antiseizure medications; PO: Postoperative Outcome(Engel's class); F: Frontal; P: Parietal; O: Occipital; T: Temporal; RF: Right Frontal; LF: Left Frontal; RP: Right Parietal; LP: Left Parietal; RP: Right Parietal; LP: Left Parietal; RO: Right Occipital; LO: Left Occipital; RT: Right Temporal; LT: Left Temporal; FAS: Focal aware seizure; FIAS: Focal impaired awareness seizures; FBTCS: Focal-to-bilateral tonic-clonic seizure; GAS: Generalized absence seizure; GTS: Generalized tonic seizure; GTCS: Generalized tonic-clonic seizure; VPA: Valproate; OXC: Oxcarbazepine; LEV: Levetiracetam; CBZ: Carbamazepine; TPM: Topiramate; NZP: Nitrazepam; LTG: Lamotrigine; PHT: phenytoin sodium; PB: phenobarbital, RT-PCR: real-time polymerase chain reaction; WB: western botting; IHC: immunohistochemistry; IF: immunofluorescence.

Table 2. Clinical and neuropathological characteristics of patients with TSC lesions.

| NO. | Sex | Disease | Surgery at Age (Years) | Age at onset of seizure | ED (Years) | Lesion | ST | SF (Months) | ASMs | PO | Application | Relative α-syn mRNA | Relative OD of α-syn protein |
|---|---|---|---|---|---|---|---|---|---|---|---|---|---|
| 1 | M | TSC | 2.0 | 0.7 | 2.0 | P | FAS | 30.0 | VGB<br>OXC<br>RPM | I | WB | | 0.74 |
| 2 | F | TSC | 2.0 | 1.0 | 1.0 | P | GTCS | 1.0 | TPM<br>VGB<br>RPM | I | IHC<br>IF | | |
| 3 | F | TSC | 3.0 | 1.0 | 2.0 | RF | GTCS | 60.0 | OXC | IV | RT-PCR<br>WB | 0.81 | 0.37 |
| 4 | F | TSC | 3.3 | 1.3 | 2.0 | LO | FAS | 45.0 | OXC<br>VPA<br>VGB | I | RT-PCR<br>WB,<br>ICH | 1.65 | 0.74 |
| 5 | F | TSC | 5.0 | 2.0 | 3.0 | LF | FIAS | 1.0 | VGB<br>LTG | I | RT-PCR<br>IHC | 6.35 | |
| 6 | F | TSC | 5.0 | 4.0 | 1.0 | RF | FIAS | 30.0 | OXC<br>LEV | I | RT-PCR | 2.24 | |
| 7 | M | TSC | 5.0 | 4.3 | 0.7 | RF | GTCS | 300.0 | OXC<br>LEV<br>TPM | I | RT-PCR<br>WB | 3.01 | 1.28 |
| 8 | M | TSC | 5.3 | 0.3 | 5.0 | T | FIAS | 15.0 | RPM<br>TPM<br>VPA<br>OXC<br>NZP | I | RT-PCR<br>WB<br>IHC | 1.39 | 0.61 |
| 9 | M | TSC | 6.0 | 5.7 | 0.3 | F | GTCS | 165.0 | VPA<br>LEV | I | RT-PCR<br>WB | 0.87 | 1.09 |
| 10 | M | TSC | 6.6 | 1.6 | 5.0 | LF | FIAS | 75.0 | RPM<br>LEV<br>OXC<br>VPA | I | RT-PCR<br>WB<br>IHC<br>IF | 1.66 | 0.55 |
| 11 | F | TSC | 7.0 | 2.0 | 5.0 | RO | GTCS | 60.0 | LEV<br>VPA<br>NZP<br>OXC | I | WB | | 0.31 |
| 12 | F | TSC | 8.0 | 3.0 | 5.0 | F | GTCS | 45 | VPA<br>LEV<br>CBZ<br>PB<br>NZP | IV | RT-PCR<br>WB | 2.11 | 1.04 |

| | | | | | | | | | | | | | |
|---|---|---|---|---|---|---|---|---|---|---|---|---|---|
| 13 | M | TSC | 9.8 | 1.8 | 8.0 | LF | GAS | 8.0 | LEV<br>OXC<br>RPM<br>VPA | I | RT-PCR<br>WB<br>ICH | 0.91 | 0.42 |
| 14 | M | TSC | 11.0 | 9.0 | 2.0 | RF | FIAS | 75.0 | VPA | I | RT-PCR<br>WB | 5.73 | 0.43 |
| 15 | M | TSC | 11.8 | 11.0 | 0.8 | LT | GTCS | 5.0 | OXC | I | WB | | 0.61 |
| 16 | M | TSC | 12.0 | 11.8 | 0.2 | LT | GAS | 1.0 | LEV | I | RT-PCR | 0.93 | |
| 17 | F | TSC | 13.0 | 6.0 | 7.0 | LP | FBTC<br>S | 3.0 | OXC<br>LTG | I | WB<br>IHC | | 0.78 |
| 18 | M | TSC | 14.0 | 9.0 | 5.0 | RF | GTCS | 4.0 | VPA | I | WB | | 0.64 |
| 19 | F | TSC | 14.0 | 12.0 | 2.0 | RF | FAS | 6.0 | OXC<br>LEV<br>CBZ | I | RT-PCR<br>WB | 1.36 | 0.64 |
| 20 | M | TSC | 15.0 | 14.8 | 0.2 | T | GTCS | 1.0 | 0XC | II | RT-PCR<br>WB | 5.06 | 0.82 |
| 21 | M | TSC | 15.0 | 5.0 | 10.0 | RF | GTCS | 4.0 | TPM<br>CBZ | III | RT-PCR<br>WB | 1.85 | 0.75 |
| 22 | M | TSC | 16.0 | 3.0 | 13.0 | F | GTCS | 10.0 | VPA<br>CBZ | I | WB | | 0.86 |
| 23 | M | TSC | 26.0 | 1.0 | 25.0 | LF | GTCS | 10.0 | OXC<br>VPA | III | RT-PCR<br>WB<br>IHC | 0.88 | 0.37 |
| 24 | F | TSC | 32.0 | 2.0 | 30 | F | GTCS | 30.0 | VPA<br>CBZ | II | WB | | 1.05 |

M=Male; F=Female; ED: Epilepsy duration; ST: Seizure type; SF: Seizure frequency; ASMs: Antiseizure medications; PO: Postoperative Outcome (Engel's class); F:Frontal; P: Parietal; O: Occipital; T: Temporal; RF: Right Frontal; LF: Left Frontal; RP: Right Parietal; LP: Left Parietal; RP :Right Parietal; LP: Left Parietal; RO: Right Occipital; LO: Left Occipital; RT: Right Temporal; LT: Left Temporal; FAS: Focal aware seizure; FIAS: Focal impaired awareness seizures; FBTCS: Focal-to-bilateral tonic-clonic seizure; GAS: Generalized absence seizure; GTCS: Generalized tonic-clonic seizure; VPA: Valproate; OXC: Oxcarbazepine; LEV: Levetiracetam; CBZ: Carbamazepine; TPM: Topiramate; NZP: Nitrazepam; LTG: Lamotrigine; PHT: phenytoin sodium; PB: phenobarbital; RPM: Rapamycin Rapamune; VGB: vigabatrin. RT-PCR: real-time polymerase chain reaction, WB: western botting; IHC: immunohistochemistry; IF: immunofluorescence.

Table 3. Clinical and neuropathological characteristics of CTX subjects.

| NO | Sex | Surgery at Age (Years) | Disease | Application | Relative α-syn mRNA | Relative OD of α-syn protein |
|---|---|---|---|---|---|---|
| 1 | M | 4 | TBI | RT-PCR<br>WB | 0.43 | 0.27 |
| 2 | M | 5 | RT glioma | RT-PCR<br>WB<br>IHC | 0.62 | 0.39 |
| 3 | M | 6 | RF primitive neuroectoblastoma | RT-PCR<br>WB | 0.51 | 0.35 |
| 4 | M | 9 | LP oligodendroglioma | RT-PCR<br>WB | 0.65 | 0.41 |
| 5 | M | 9 | RF primitive neuroectoblastoma | WB | | 0.67 |
| 6 | M | 11 | LT glioma | WB | | 1.41 |
| 7 | M | 11 | TBI | RT-PCR<br>WB | 1.20 | 0.66 |
| 8 | M | 12 | LP ependyblastoma | RT-PCR<br>WB | 0.98 | 1.10 |
| 9 | M | 13 | RT meningioma | RT-PCR<br>WB<br>IHC | 1.04 | 0.66 |
| 10 | M | 14 | RT glioma | WB<br>IHC | | 0.47 |
| 11 | F | 15 | LT glioma | WB | | 1.30 |
| 12 | M | 16 | LF glioma | WB<br>IHC | | 0.77 |
| 13 | F | 16 | LP glioma | WB | | 1.01 |
| 14 | M | 17 | LF glioma | WB | | 1.19 |
| 15 | F | 19 | RT glioma | WB | | 0.98 |
| 16 | M | 20 | TBI | WB<br>IHC<br>IF | | 0.69 |
| 17 | M | 21 | RT glioma | WB | | 1.06 |
| 18 | M | 22 | LTP meningioma | WB<br>IHC<br>IF | | 0.86 |
| 19 | F | 25 | RF glioblastoma | RT-PCR<br>WB | 1.46 | 0.77 |
| 20 | F | 25 | LTP meningioma | WB<br>IHC | | 1.15 |
| 21 | M | 26 | LF glioblastoma | WB | | 0.52 |

| | | | | | | |
|---|---|---|---|---|---|---|
| 22 | F | 27 | PO meningioma | RT-PCR<br>WB<br>IHC | 1.25 | 0.73 |
| 23 | F | 28 | Right primitive neuroectoblastoma | WB | | 3.27 |
| 24 | M | 35 | RF oligodendroglioma | WB | | 1.49 |
| 25 | M | 35 | RF oligodendroglioma | WB | | 2.03 |
| 26 | F | 37 | LT glioma | RT-PCR<br>WB | 1.85 | 1.51 |

RF: Right Frontal; LF: left frontal; RP: right parietal; LP: left parietal; RP: Right Parietal; LP: Left Parietal; RO: Right Occipital; LO: Left Occipital; RT: Right Temporal; LT: Left Temporal; LTP: Left Temporal Parietal; PO: Parietal Occipital; TBI: Traumatic Brain injury, RT-PCR: real-time polymerase chain reaction, WB: western botting; IHC: immunohistochemistry; IF: immunofluorescence. Average OD value of the sample was adopted if it was used as control for both FCD IIb and TSC lesions in two independent experiments.
